# Cytostretch, an Organ-on-Chip Platform

**DOI:** 10.3390/mi7070120

**Published:** 2016-07-14

**Authors:** Nikolas Gaio, Berend van Meer, William Quirós Solano, Lambert Bergers, Anja van de Stolpe, Christine Mummery, Pasqualina M. Sarro, Ronald Dekker

**Affiliations:** 1 Laboratory of Electronic Components, Technology & Materials (ECTM), DIMES, Delft University of Technology, 2628 CD Delft, The Netherlands; w.f.quirossolano@tudelft.nl (W.Q.S.); p.m.sarro@tudelft.nl (P.M.S.); ronald.dekker@philips.com (R.D.); 2 Department of Anatomy and Embryology, Leiden University Medical Center, Leiden 2333 ZC, The Netherlands; b.j.van_meer@lumc.nl (B.v.M.); C.L.Mummery@lumc.nl (C.M.); 3 Department of Dermatology, VU University Medical Center Amsterdam, Amsterdam 1081 HT, The Netherlands; l.bergers@vumc.nl; 4 Philips Research, Eindhoven 5656 AE, The Netherlands; anja.van.de.stolpe@philips.com

**Keywords:** organ-on-chip, customizable, modular, platform, Cytostretch, micro-electrode array, micro-grooves, through-membrane pores, strain gauges, stem cells

## Abstract

Organ-on-Chips (OOCs) are micro-fabricated devices which are used to culture cells in order to mimic functional units of human organs. The devices are designed to simulate the physiological environment of tissues in vivo. Cells in some types of OOCs can be stimulated in situ by electrical and/or mechanical actuators. These actuations can mimic physiological conditions in real tissue and may include fluid or air flow, or cyclic stretch and strain as they occur in the lung and heart. These conditions similarly affect cultured cells and may influence their ability to respond appropriately to physiological or pathological stimuli. To date, most focus has been on devices specifically designed to culture just one functional unit of a specific organ: lung alveoli, kidney nephrons or blood vessels, for example. In contrast, the modular Cytostretch membrane platform described here allows OOCs to be customized to different OOC applications. The platform utilizes silicon-based micro-fabrication techniques that allow low-cost, high-volume manufacturing. We describe the platform concept and its modules developed to date. Membrane variants include membranes with (i) through-membrane pores that allow biological signaling molecules to pass between two different tissue compartments; (ii) a stretchable micro-electrode array for electrical monitoring and stimulation; (iii) micro-patterning to promote cell alignment; and (iv) strain gauges to measure changes in substrate stress. This paper presents the fabrication and the proof of functionality for each module of the Cytostretch membrane. The assessment of each additional module demonstrate that a wide range of OOCs can be achieved.

## 1. Introduction

Research and development (R&D) expenditure by the pharmaceutical industry has increased exponentially since the 1970s and has become a large share of their overall cost structure. However, this has not led to an increase in the number of drugs entering the market, explaining in part why average development costs per drug have risen to an estimated $2.6 billion [1]. The consequence is an urgent need for new technologies to identify and validate reliable new human therapeutic targets, and for more effective drug development [2].

In current preclinical drug R&D, cell cultures and animal models are often used to predict responses to drug compounds in humans. However, these models do not always capture human physiology and pathology sufficiently to be used as good surrogates [3]. One alternative under investigation is the use of primary human cells or stem cell line–derived cells in culture but it is clear that to be true substitutes, other aspects of their (patho) physiology in situ need to be considered. This may include cell and tissue geometries, electrical activity and substrate mechanics. 

Organ-on-Chips (OOCs) are models which take these considerations into account by combining sophisticated chip technology with biology. This enables human responses to be recapitulated in vitro more accurately than in other systems described so far [2,3]. OOCs are micro-fabricated devices comprised of microfluidic parts, e.g., channels and chambers in plastic foils, and a stretchable or porous membrane, on which cells are cultured. OOCs make it possible to culture complex tissue structures at a small scale [4]. The cultured cells, often different cell types, are allowed to interact in vitro to create a tissue model of functional units of an organ. These may, for example, be nephrons of the kidney, alveoli of the lungs, or the blood-brain barrier of the vascular system. Growth, proliferation, differentiation, maturation and controlled interactions between different cell types in the model are facilitated in the controlled environment of the OOC chip device [4]. 

Most OOCs are fabricated using polydimethylsiloxane (PDMS). This material has several advantages: low cost, easy patterning via soft lithography, biocompatibility, optical transparency and permeability to gasses [5,6]. Often, OOCs are fabricated by sealing three-dimensional (3D) PDMS structures patterned through replica molding processes, also known as soft lithography [6]. This technique allows simple and rapid modeling of new OOC devices and is therefore particularly useful in rapid testing of new designs [2]. However, soft lithography is based on labor-intensive procedures that reduce device throughput and yield, a hurdle to large-scale fabrication. This conflicts with the need for low-cost OOCs for cell culture which is conventionally based on the extensive use of disposables. 

Research to date has focused on the development of devices that represent a single functional unit of an organ. There are thus well-described OOCs for liver [7], kidney [8], lung [9], and gut [10], among multiple other organs [2]. Some developments realized multiple functionalities in one OOC. For example, Booth et al. [11] presented a blood-brain barrier model composed of a microfluidic system, a filter membrane, which supports and separates multiple cell cultures, and two electrodes, which characterize the membrane impedance. Furthermore, Huh et al. [9] presented a microfluidic device with a stretchable cell-culture substrate embedded in a microfluidic channel to apply hemodynamic shear stress and mechanical strain to a lung cell culture. However, due to the fabrication techniques used for these OOCs, including other functionalities requires a complete redesign of the system and complicates the fabrication. Moreover, these systems are usually not compatible with previously developed silicon-based sensors such as thermal, optical, pH and electrical sensors [2,3]. Eliminating these drawbacks would greatly facilitate progress in OOC development.

**Figure 1:**
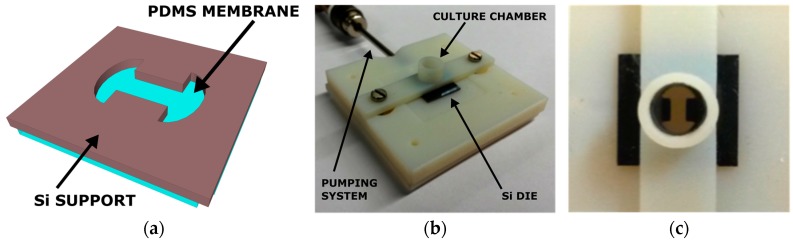
(**a**) Back view of the 3D sketch of the Cytostretch; (**b**) Example of a 3D-printed holder for the Cytostretch, including a cell culture chamber on top of the die and a pumping system to inflate the membrane; (**c**) Top view of the device mounted in the holder.

Here, we report a novel customizable membrane platform, also called Cytostretch, based on conventional cleanroom-compatible micro-fabrication processes allowing wafer-scale fabrication and integration of silicon-based electronics. This enables large-quantity fabrication of a stretchable membrane with different features, e.g., micropores, micro-grooves, electrodes, so that it may be incorporated in different OOCs for different cell culture applications. The platform builds on the technology originally developed for a heart-on-chip model by Pakazad et al. [12]. The basic element of the Cytostretch membrane platform consists of a freestanding PDMS membrane, shaped as a dog-bone and fabricated on a silicon chip, as shown in Figure 1a. This chip was attached to a plastic cylinder or 3D-printed holder to contain cell culture medium, as shown in Figure 1b,c, thus enabling cell research. We envision that by adding different features to the membrane, adapting the shape and dimensions, and incorporating it into different microfluidic devices, it could be used for different OOCs. The features could be added through fabrication modules, which could be readily added or removed from the processing without affecting the fabrication complexity. In this way it is possible to customize the membrane to realize specific, more complex functionalities in OOCs while still yielding numerous devices. 

In this paper we present the Cytostretch modules developed to date. The main goal of this paper is to show the potential of this approach by proving the functionality of each module. These are individually described by presenting their integration in the Cytostretch membrane and their characterization in a particular OOC application.

## 2. Cytostretch Membrane Product Platform

The Cytostretch PDMS membrane is the carrier for the other technology modules. PDMS membranes have two main advantages as substrates for stem cell culture. Firstly, substrate stiffness can be tuned by altering the ratio between the elastomer and cross-linker [13]; this can modulate cell morphology, function, and fate [14]. Previous work suggests that matching the substrate elasticity with in vivo tissue elasticity can induce and direct stem cell differentiation [13]. For example, soft substrates promote neuronal differentiation, while substrates with bone-like stiffness promote osteogenic differentiation [13].

Secondly, the elasticity of PDMS allows the membrane to be inflated and deflated statically and cyclically, using a pneumatic system; this exerts physical forces on cells cultured on the membrane. Hemodynamic shear stress or physical forces have, for example, been reported to affect the developmental fate of human pluripotent stem cells (hPSC) in culture [13,15] and stress induced by mechanical deformation might reveal disease-related functional changes. For example, cell spreading, growth and morphology can be promoted by cyclically stretching the culture substrate [16,17]. 

**Figure 2:**
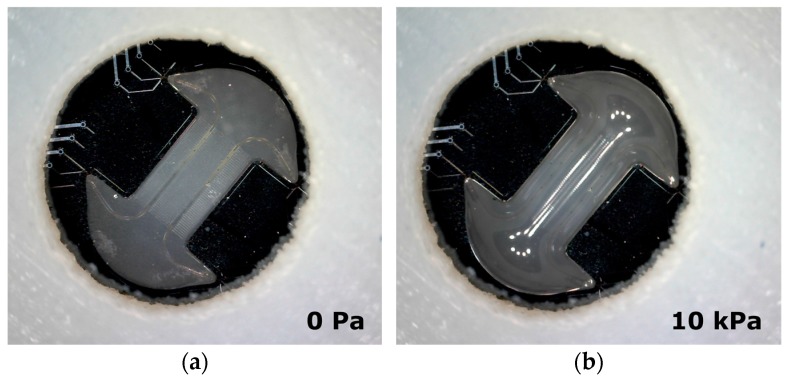
(**a**) Cytostretch (with MEA module embedded) at relaxed state; (**b**) The Cytostretch device during inflation by applying an air pressure of 10 kPa on the back of the membrane.

Figure 2 shows Cytostretch membrane in a relaxed state (a) without air pressure and (b) bi-axially expanded with air pressure (10 kPa) applied. In this situation cells can only be cultured on the side of the membrane that is covered with culture fluid, and not on the air-exposed side. The Cytostretch membrane has been previously stretched with pressure values up to 40 kPa, with correspondent vertical displacements of 500 μm [12].

### Fabrication

**Figure 3:**
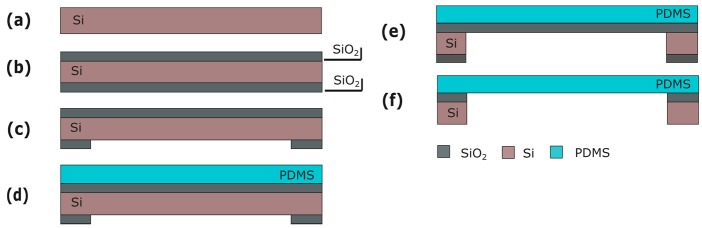
Process flow for the product platform: (**a**) Si wafer; (**b**) PECVD SiO_2_ deposition; (**c**) Back SiO_2_ patterning; (**d**) PDMS deposition; (**e**) DRIE Si etching; (**f**) Wet SiO_2_ etching. Figures are not to scale.

Silicon (Si) wafers are used as a starting substrate for the fabrication of the Cytostretch membrane platform. Processing starts with the deposition of 2 and 6 μm of silicon oxide (SiO_2_) by plasma-enhanced chemical vapor deposited (PECVD) on the front and back of the wafer, respectively (Figure 3b). The SiO_2_ layer on the back is patterned by dry-etching in order to define the membrane area (Figure 3c). Subsequently, a 15-μm-thick PDMS layer is spun onto the front of the wafer at 3500 rpm for 30 s and cured for 30 m at 90 °C (Figure 3d). Finally, the membrane is released by removing the Si and the SiO_2_ layers from underneath the membrane using deep reactive ion etching (DRIE) and buffered hydrofluoric acid (BHF), respectively (Figure 3f). 

## 3. Through-Membrane Micro-Pores

A Cytostretch membrane with through-membrane micro-pores of defined size may be useful for applications where a cell layer or 3D tissue needs to be supported while allowing the exchange of biological signals through the membrane from one chamber of a microfluidics device to a second chamber. An example of such signaling is immune cell migration from blood vessels to a wound in the skin [18,19]. Conventionally, commercially available low-porosity non-stretchable thin foils (*t*~10–20 μm) are used. However, a highly porous support is preferred in this type of application so that the membrane is optimally permeable for the signal. 

Stretchability might be useful for investigating the role of mechanical load in skin scarring [20]. The utility of a Cytostretch membrane with defined pore size is illustrated in Section 3.3 by the migration of THP-1 monocytes (an immune cell-line) in a chemotactic essay.

### 3.1. Fabrication

**Figure 4:**
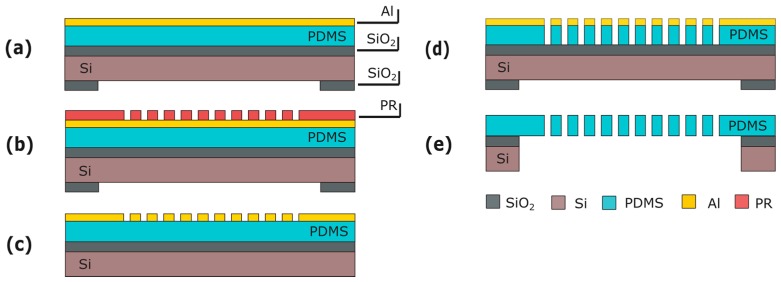
Process flow for the through-membrane micro-pore array: (**a**) Al sputtering; (**b**) PR spinning and patterning; (**c**) Al patterning; (**d**) PDMS patterning; (**e**) Membrane releasing. Figures are not to scale.

This module consists of an array of through-membrane micro-pores patterned in the PDMS layer. To include the pores, this module is inserted into the fabrication flow immediately after PDMS spinning and curing (Figure 3d). An aluminum (Al) layer is sputtered on the PDMS at room temperature (Figure 4a). The Al is masked with 4 μm of photoresist (PR) (AZ ECI 3027) and dry-etched (Figure 4b,c). The lithography and etching processes used are optimized to circumvent issues caused by the difference between the expansion coefficients of the PDMS and the PR. Besides serving as a hard mask, the Al layer reduces the effects of the differences in expansion coefficients by acting as a buffer layer between PDMS and PR. Next, the pore array is dry-etched in the PDMS layer (Figure 4d) and the Al hard-etch mask is removed by wet-etching. The fabrication ends by releasing the membrane as seen in Section 2. (Figure 4e).

### 3.2. Characterization 

**Figure 5:**
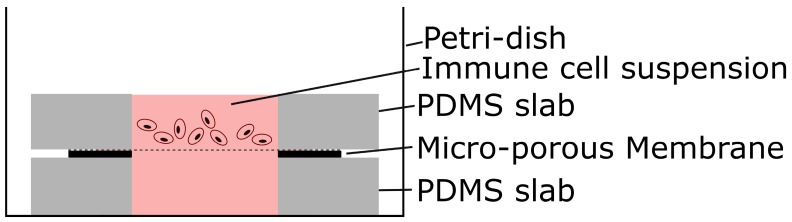
Schematic of setup for simple migration experiment.

The resulting through-membrane micro-pores were characterized after fabrication using scanning electron microscopy (SEM). THP-1 cells, cultured in RPMI-1640 medium, were used for the immune cell migration experiment. A basic test similar to a standard Boyden chamber experiment [21] was performed (Figure 5). The membrane was placed between two PDMS slab with a center hole of 5 mm to create two fluid chambers. The bottom chamber was filled with culture medium, which was enriched with monocyte chemoattractant protein 1 (MCP-1) while the immune cells were seeded in the top chamber. The MCP-1 provided a strong stimulus to attract monocytes. Images of the membrane and the volume below were recorded with a Leica inverted phase microscope, 20× objective, at 5 min and 3.5 h after seeding the membrane.

### 3.3. Results and Discussion

**Figure 6:**
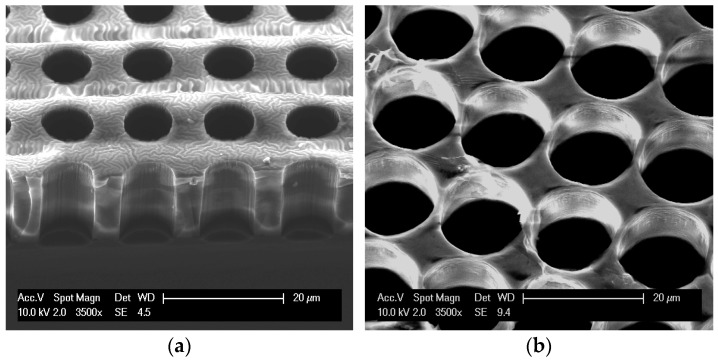
SEM images of microporous PDMS membranes: (**a**) 15-µm-thick membrane with through-membrane pores 8 µm in diameter; (**b**) 9-µm-thick membrane with through-membrane pores 14 µm in diameter.

**Figure 7:**
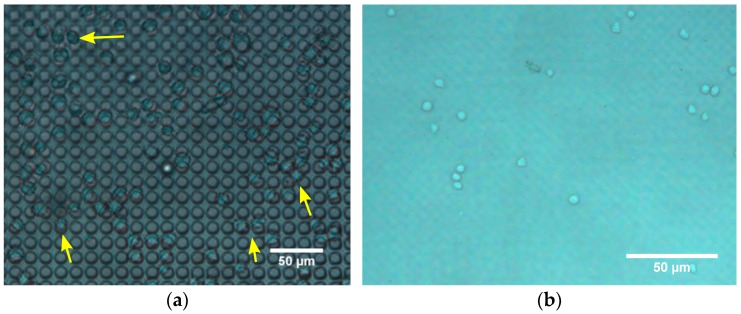
Phase contrast images of migration experiment. (**a**) The top of the micro-porous membrane 5 min after seeding shows immune cells resting on pores; (**b**) The volume below the membrane 3.5 h after seeding shows immune cells floating.

Pores 8–14 µm in diameter (and 15 µm pitch) were etched in the Cytostretch membrane. The SEM pictures of a membrane patterned with pores with an 8 µm diameter and a 14 µm diameter are shown in Figure 6a,b, respectively. For the migration experiment, membranes with 8 µm pores were used. After 5 min, cells were observed on top of the membranes, settling on/near the pores (Figure 7a). At that time, cells were not observed on the bottom of the membrane. After 3.5 h, however, the immune cells were visible in the fluid below the membrane (Figure 7b) and had virtually disappeared from the top of the membrane. This shows that the membrane does not restrict migration to the opposite side.

## 4. Micro-Electrode Array

The activity of electrically active cells, such as heart, muscle and neural cells, can be recorded on microelectrode arrays (MEAs). Changes in this electrical response due to drugs have shown to be predictive for their pharmacological safety profile [22]. MEAs can also be used to stimulate electrogenic cells, and modulate their behavior by means of electrical impulses [23]. Furthermore, electrical stimulation might play a role in the cardiac differentiation of human embryonic stem cells [24]. 

A stretchable electrode-containing Cytostretch membrane can be inserted into culture devices [12,25]. The stretchable membrane consists of an array of 12 titanium nitride (TiN) electrodes embedded in the PDMS membrane. In order to demonstrate the functionality of the module, the Cytostretch membrane was tested by recording the electrical activity of human pluripotent stem cell–derived cardiomyocytes (hPSC-CMs) plated on the Cytostretch membrane.

### 4.1. Fabrication

**Figure 8:**
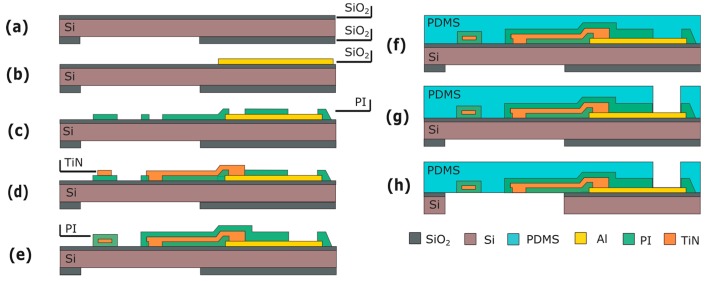
Process flow for the MEA module: (**a**) Substrate; (**b**) Al deposition and patterning; (**c**) First layer of polyimide (or, alternatively, parylene) is spun and patterned; (**d**) TiN deposition and patterning; (**e**) Second layer of polyimide (or, alternatively, parylene) is spun and patterned; (**f**) PDMS deposition; (**g**) PDMS patterning; (**h**) Membrane releasing. Figures are not to scale.

This module is fabricated before the PDMS deposition (Figure 3d). The processing starts by depositing and patterning 500 nm of Al on the front side of the wafer (Figure 8b). This metal layer is used for the contact pads and the electrical interconnects outside the membrane area. Next, the metal lines extending from the contact pads to the electrodes are fabricated. These consist of a 100-nm-thick layer of sputtered TiN, sandwiched between two layers of polymide or, alternatively, parylene (Figure 8e). 

Next, PDMS is spin-coated and cured (Figure 8f), and metal openings to the electrical contacts are etched through the PDMS layer on the front of the wafer (Figure 8g), using an Al layer as a hard mask as done in the previous module. The process flow again ends with the release of the membrane as previously described (Figure 8h).

### 4.2. Characterization

The Cytostretch membrane equipped with the MEA was characterized after microfabrication using SEM. At the current stage the MEA has only been tested for recording the electrical activities of cells. To investigate the electrical recording capabilities of the electrodes, hPSC-CMs were plated onto Cytostretch with the MEA module. First, the devices were sterilized in ethanol and coated with Matrigel (Invitrogen, Carlsbad, CA, USA). Next, the cells were plated and cultured on the device for 36 h in a CO_2_ incubator at 37 °C. The readout of the field potential of the hPSC-CMs is performed with a Multi Channel System (MCS) USB-MEA-System. Cytostretch interfaces with this system through a printed circuit board as presented in [12,25]. 

### 4.3. Results and Discussion

**Figure 9:**
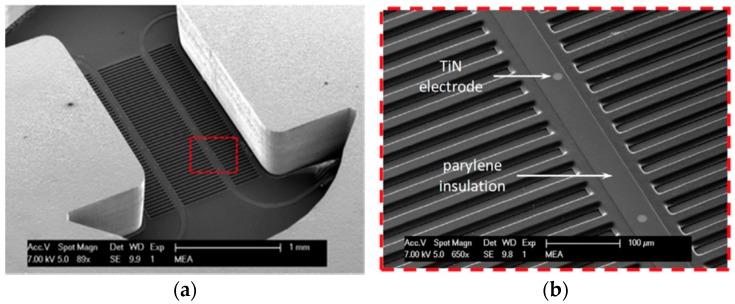
(**a**) SEM image of the Cytostretch chip from the back; (**b**) Close-up of the area highlighted in (**a**) depicting transversal micro-grooves, the exposed TiN electrodes and parylene insulation of the metal tracks. Adapted from [25].

**Figure 10:**
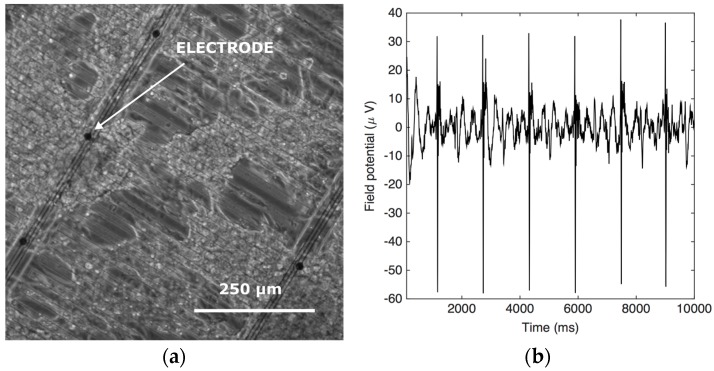
(**a**) Optical images of cardiac induced pluripotent stem cell (iPSC) on the Cytostretch device; (**b**) The field potential recording from one of the electrodes.

SEM images of the Cytostretch device including both the MEA and the micro-groove modules are shown in Figure 9a,b. Twelve electrodes with a minimum geometric surface area (GSA) of 50 µm^2^ can be fabricated, although, for this application, electrodes with a 110 µm^2^ GSA were chosen. The maximum number of electrodes included in the Cytostretch membrane is defined by the width of the interconnect tracks in the dog-bone-shaped membrane, as presented by Pakazad et al. [12,25]. The hPSC-CMs plated on the Cytostretch started beating spontaneously within three days, demonstrating their viability and functionality. Figure 10a shows the Cytostretch with hPSC-CMs after three days of culture. 

The recorded signal shows the electric field potential of the hPSC-CMs on top of the electrode. The signal was filtered with Matlab using a band pass filter (2–200 Hz) and a cut-off filter (50 Hz) to remove motion artifacts. The spikes in the electric field potential seen in Figure 10b correspond to the depolarization phase of the action potential [25]. Typical recorded signals are in the order of 100 μV. The signal has a relatively low signal-to-noise ratio (SNR) compared to other works [26,27], due to the high electrochemical impedance of the flat and subcellular electrodes included in the Cytostretch device [12,25]. The impedance can be improved by coating the electrodes with porous coating such as carbon nanotubes as shown by the author in [28].

## 5. Micro-Grooves 

Organ tissues in situ are highly organized, layered structures that incorporate multiple cell types to simulate complex organ-specific functions, including organ repair by cell renewal, vascularization and inflammatory responses. Both macroscopic and microscopic organization of cells takes place so that, for example, stem cells of the intestine orient themselves in the intact tissue and smooth muscle cells within the intestine that generate the peristaltic contraction, co-integrate appropriately. In striated muscle, anisotropic orientation of cells is crucial, since it determines the direction in which the muscle shortens. Feinberg et al. showed anisotropic cardiac myocyte alignment by micro-contact printing of an extracellular matrix (ECM) protein [29]. However, this manual technique is not suitable for micro-fabrication and is cumbersome in a cell lab.

The Cytostretch membrane can be patterned to create micro-grooves to control the orientation of cells in the culture. This module was employed to provide topological cues to hPSC-CMs and assess their anisotropic alignment after seven days in culture.

### 5.1. Fabrication

**Figure 11:**
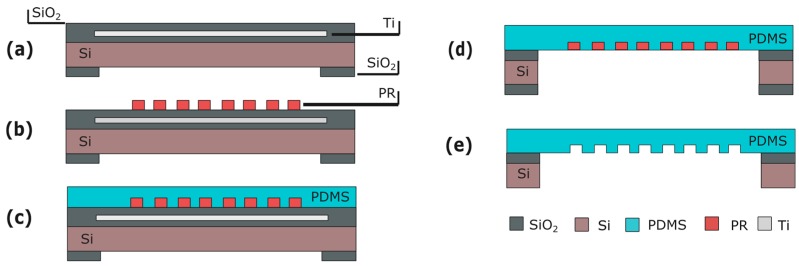
Process flow for the micro-groove module: (**a**) Substrate with Ti mask embedded in the front-side SiO_2_ layer; (**b**) PR spinning and patterning; (**c**) PDMS patterning; (**d**) Membrane releasing; (**e**) PR stripping. Figures are not to scale.

The micro-grooves are fabricated on the same side of the Cytostretch membrane as the MEA electrodes are deposited to determine electrical activity in organized (cardiac) tissues. The micro-groove module is therefore fabricated before spinning on the PDMS. This module requires a layer of titanium (Ti) embedded in the SiO_2_ substrate (Figure 11a). This masks the PR from UV light generated during final DRIE etching, preventing unwanted PR cross-linking. After substrate fabrication (Figure 11a), 4-μm-thick PR is spin-coated and patterned (Figure 11b). The PR will serve as a mold for the micro-pattern formation in the PDMS membrane. Next, PDMS is spun onto the wafer and cured (Figure 11c) and the membrane is released (Figure 11d). After etching the oxide and Ti UV block layer, the PR mold is dissolved in acetone.

### 5.2. Characterization

The micro-grooves were characterized using SEM. In order to assess the degree of alignment, the micro-patterned PDMS was UV-treated and coated with Matrigel (Invitrogen). Commercially available human induced pluripotent stem cell (hiPSC)-derived cardiomyocytes (Pluriomics, Galileiweg, The Netherlands) were thawed and after seven days replated onto the micro-grooved PDMS. Cells were cultured in Pluriomics Cardiomyocyte Medium. 

After seven days in culture on the micro-patterned substrate, the cells were fixed with 2% paraformaldehyde and stained with an anti-alpha-actinin antibody, anti-troponin-I antibody and DAPI (4',6-diamidino-2-phenylindole) to reveal the sarcomeric structures and cell nucleus. 

### 5.3. Results and Discussion

A SEM picture of the micro-grooves embedded in the Cytostretch membrane is shown in Figure 9a,b. With this fabrication technique, the width and the height of the micro-grooves are determined by the lithography step shown in Figure 11b. The width can range from 0.5 μm to hundreds of μm, while the depth of the grooves can range from 1 to 12 μm. hPSC-CMs plated on standard culture substrates such as culture plastic or glass coverslips show a more isotropically orientated sarcomeric organization compared to cardiomyocytes in vivo, which is thought in part to be regulated by their shape [30]. hPSC-CMs are less mature than adult myocardium and this is evident in several of their physiological properties, among which cardiomyocyte elongation: adult cardiomyocytes have a length-to-width ratio of 5–9.5 to 1 whereas hPSC-CMs are limited to a ratio of 3 to 1 [31]. 

**Figure 12:**
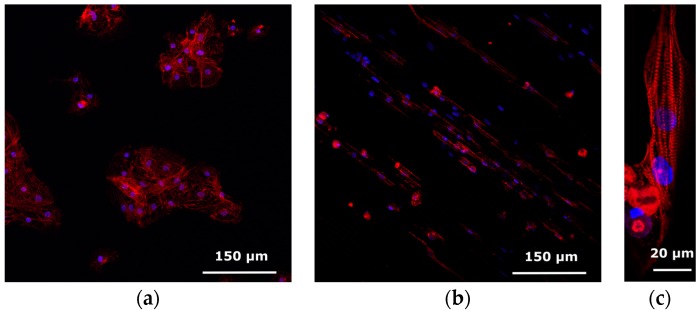
Confocal images of hPSC-CMs stained for anti-alpha-actinin (red) and DAPI (blue) to reveal the sarcomeric structure and cell nucleus. (**a**) hPSC-CM on plain PDMS; (**b**,**c**) hPSC-CM on micro-patterned PDMS.

Compared to hPSC-CMs cultured on plain PDMS-coated coverslips (Figure 12a), the topological cues of the micro-patterned PDMS increase the aspect ratio of hPSC-CMs and induce a more rectangular shape, possibly by limiting cell width. Figure 12b,c show, respectively, a large-field view of the cell culture grown on the micro-grooves and a representative hPSC-CM cultured on micro-grooved PDMS with an aspect ratio of 7:1, indicating a more physiological cardiomyocyte elongation.

## 6. Strain Gauges

To date, most OOCs have used static optical techniques (immunofluorescence end-point detection, microscope cell imaging) to measure the deformation of PDMS membranes during inflation [32]. In a previous work we presented a novel Cytostretch membrane module that integrates sensing structures into the membrane to quantify the strain applied to heart or muscle cells [33], or exerted by them during contraction. Ti strain gauges were integrated in the Cytostretch PDMS membrane, and characterized by measuring the resistance change as a result of the applied strain. 

### 6.1. Fabrication

**Figure 13:**
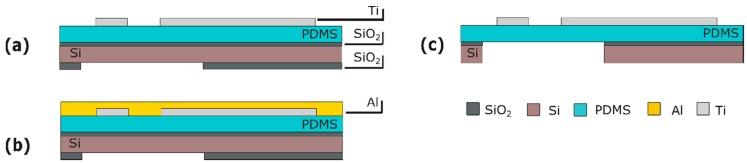
Process flow for the strain gauges: (**a**) Ti deposition and patterning; (**b**) Al deposition; (**c**) membrane releasing and Al etching. The figures are not to scale.

A 100-nm-thick layer of titanium (Ti) is deposited by sputter coating onto the PDMS layer at room temperature to avoid cracking of subsequent layers. The Ti layer is patterned to form strain gauges with different geometries by dry etching with 3-µm-thick positive PR as the masking layer (Figure 13a). To protect the strain gauges during release etch from the back, the front of the wafer is temporarily covered with a 200-nm-thick layer of Al (Figure 13b). After the membrane is released, the Al layer is selectively removed in a solution of acetic, phosphoric and hydrofluoric acid without damaging the strain gauges (Figure 13c). 

### 6.2. Characterization

**Figure 14:**
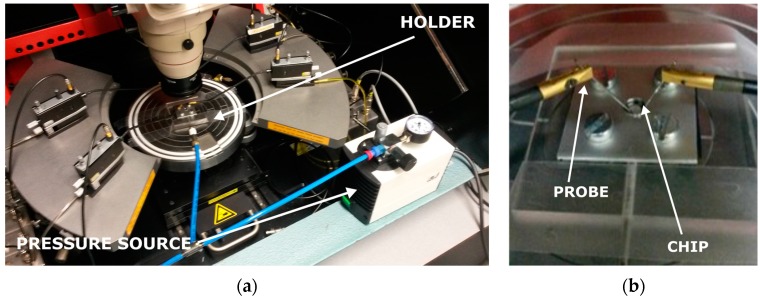
Measurement setup (**a**) and custom-made holder (**b**) used to measure the resistance change of the Ti strain gauges in the Cytostretch membrane platform.

The strain gauges were characterized in a custom-made setup to test their resistance under mechanical strain. The setup consists of a probe station, a custom-made holder for the Cytostretch and a pressure source (Figure 14a,b). To verify the change in resistance of the gauge when the membrane is deformed, the probe station is configured to continuously record the I–V characteristics of the devices. The deformation of the membrane is induced by applying a positive pressure to the membrane through the cavity formed by the silicon and the bottom of the holder. The pressure change is monitored using a standard manometer capable of measuring pressures down to 100 Pa.

### 6.3. Results and Discussion

**Figure 15:**
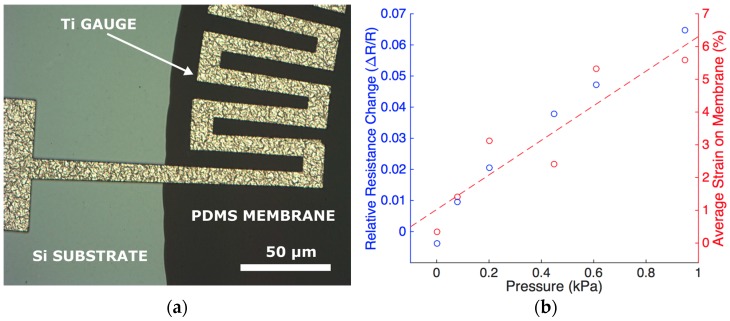
(**a**) Optical image from the front side showing a close-up of the Ti gauges at the interface between the silicon substrate and the PDMS membrane. As presented in [33], the strain gauges were fabricated on circular membranes; (**b**) Relative resistance change of a strain gauge (primary *Y* axis) and the average strain on the membrane (secondary *Y*-axis) as function of pressure.

Figure 15a shows a Ti strain gauge fabricated on the edge of the Cytostretch PDMS membrane. When the membranes are stretched by applying a controllable pressure up to 1 kPa, the relative resistance change is approximately 5% (Figure 15b). This can be related to the strain in the membrane obtained analytically, as observed in the secondary axis [32]. As mentioned in Section 2 strain can be used to induce specific stem cell fate. Hence, the membrane acts as the tunable substrate for cell culture and the strain gauges make it possible to continuously monitor the strain applied to the Cytostretch membrane. The characterization of the device demonstrates the feasibility of sensor integration in the Cytostretch membrane platform.

## 7. Conclusions 

Although OOCs in combination with appropriate cells and microenvironments are promising tools for disease modeling, drug efficacy and toxicity tests, their low technological readiness is a hurdle for commercialization, large-scale production and fabrication compatibility with silicon-based sensors. Here, a modular and customizable membrane platform for OOCs is presented. The membranes are designed and fabricated with conventional integrated circuit (IC) and microelectromechanical systems (MEMS) technologies, to enable high-yield, low-cost volume production and the integration of standard silicon-based sensors and actuators.

The platform consists of a micro-fabricated PDMS membrane, with four different technology membrane variants that can be independently inserted in the main fabrication flow without affecting other features to add functionality depending on the requirements of a particular application. The membrane variants have been described in detail and their fabrication process has been discussed. Moreover, the functionality of each membrane has been demonstrated in a typical OOC application.

The first technology module is used to etch an array of through-membrane pores in order to create a PDMS membrane with pores of a defined size that enables signal exchange through the membrane. The membrane was tested by studying the migration of immune cells through the pores. The second module consists of an integrated stretchable micro-electrode array. The electrode array was tested by measuring the electric field potential of cardiomyocytes cultured on the device’s membrane under cyclic stretch. The third technology module adds micro-grooves, molded in the PDMS membrane. The grooves are used to align cells and improve the orientation and sarcomeric organization of hPSC-CMs cultured on the membrane. The last module adds strain gauges that are used as a feedback sensor in order to enable real-time measurement of the membrane strain.

The modular character of the Cytostretch membrane platform makes it highly suitable for realizing various functions in OOCs when integrated with appropriate microfluidics. New technology modules will be developed in the near future to present more features and functions, all allowing large-scale OOC membrane manufacture. This could provide new opportunities for the field of OOCs.

## Abbreviations

The following abbreviations are used in this manuscript:

OOC: Organ-on-Chip
PDMS: Polydimethylsiloxane
hPSC: Human Pluripotent Stem Cell
hPSC-CM: hPSC Derived Cardiomyocytes
SEM: Scanning Electron Microscopy
MEA: Micro-Electrode Array
ECM: Extra Cellular Matrix
PR: PhotoResist
